# Effects of modified Danggui Sini Decoction as adjuvant therapy for angina pectoris in coronary heart disease: a systematic review and meta-analysis based on randomised controlled trials

**DOI:** 10.3389/fphar.2024.1375795

**Published:** 2024-06-04

**Authors:** He Wang, Changxing Liu, Xinyi Guo, Jianfei Yang, Yabin Zhou

**Affiliations:** ^1^ The First Hospital of Heilongjiang University of Chinese Medicine, Harbin, China; ^2^ First Clinical College of Medicine, Heilongjiang University of Chinese Medicine, Harbin, China; ^3^ Clinical College of Medicine, Chengdu University of Traditional Chinese Medicine, Chengdu, China

**Keywords:** modify Danggui Sini Decoction, conventional western medicine, angina pectoris, meta-analysis, systematic review

## Abstract

**Introduction:**

This systematic review evaluates the efficacy of the Chinese herbal formula modified Danggui Sini Decoction as an adjunctive treatment for angina pectoris in patients with coronary heart disease.

**Methods:**

We conducted a comprehensive search for randomized controlled trials that investigated the effects of modified Danggui Sini Decoction in combination with conventional Western medication on angina pectoris in coronary artery disease, published up to July 2023 across eight databases, including China Knowledge International Literature screening and data extraction were performed by two researchers following predefined inclusion and exclusion criteria. The quality of included studies was assessed using the Cochrane Handbook version 5.1, and meta-analysis was executed via RevMan 5.4 software.

**Results:**

Thirteen studies encompassing 1,232 participants were incorporated. The meta-analysis revealed that combining modified Danggui Sini Decoction with conventional Western medication significantly enhanced overall clinical efficacy, reduced the duration of angina attacks, decreased the Chinese medicine syndrome score, improved inflammatory markers and cardiac function, lowered serum NT-proBNP levels, and elevated the Seattle Angina Questionnaire scores compared to the control group.

**Conclusion:**

Modified Danggui Sini Decoction, when used alongside conventional Western medications, shows promise in treating coronary artery disease patients with angina pectoris and may serve as a beneficial adjunctive therapy in clinical settings. Nonetheless, due to the limited quantity and quality of the included studies, further high-caliber research is essential to substantiate these findings.

**Systematic Review Registration:**

https://inplasy.com/? s=202390078, identifier INPLASY 202390078.

## 1 Introduction

Coronary arteriosclerotic heart disease, also known as coronary heart disease (CHD), is a cardiac condition caused by atherosclerotic lesions in the coronary arteries. This leads to narrowing and blockage of the vessel lumen, resulting in myocardial ischemia or necrosis (Guo Z. et al., 2023). Angina pectoris, characterized by transient myocardial ischemia and chest pain, is the most common clinical manifestation of CHD and can progress to acute myocardial infarction with a high mortality rate (Wang and Chen, 2018). A global burden of disease study in 2017 reported 126.45 million cases of ischemic heart disease worldwide, including 10.6365 million new cases, leading to 8.9304 million deaths and making it the leading cause of mortality globally (Dalys and Collaborators, 2018; GBD Causes of Death Collaborators, 2018). Further research has indicated that the ratio of years lived with disability (YLD) to years of life lost (YLL) due to CHD in China is 14.2:1, with the burden of CHD ranking second and an absolute value increase of 122.0% (China Cardiovascular Health and Disease Report, 2021 Coronary Heart Disease Section, 2022; Buyiman et al., 2023). Consequently, there is a pressing need to develop early, safe, and cost-efficient interventions to improve patient daily functioning, enhance their quality of life, and alleviate the economic burden on individuals and society.

Currently, the treatment goals for CHD aim to reduce the frequency and severity of angina attacks, and prevent cardiovascular events such as acute myocardial infarction (AMI), sudden cardiac death, and heart failure (Xiaoping and Zhao, 2023). Antiplatelet aggregating drugs are commonly used in Western medicine, with severe cases possibly requiring percutaneous coronary intervention (PCI) or coronary artery bypass grafting (CABG) (Yassen et al., 2023). However, despite significant improvements from interventional therapies and conventional Western medicine, the long-term efficacy in improving survival rates and decreasing recurrence rates of CHD remains unclear, with considerable side effects (Guo J. et al., 2023). CHD not only threatens the health and life of patients but also imposes a significant economic burden on society and families. Among cardiovascular diseases, the recurrence rate of coronary angina is notably high, severely affecting patients’ normal life and work (Jia-Yi and Xi-Ping, 2023). Therefore, an increasing number of researchers are turning their focus to complementary and alternative medicine.

In traditional Chinese medicine (TCM), CHD and angina pectoris are categorized under “chest impediment” and “heartache.” The etiology of these conditions is attributed to deficiencies in Qi, blood, Yin, and Yang as underlying factors, complemented by Qi and blood stasis, cold coagulation in the blood vessels, and phlegm obstruction as primary manifestations (Liu et al., 2023). The modified Danggui Sini Decoction, derived from the “Shang Han Lun,” is reputed to nourish and activate blood, warm meridians, and disperse cold. Recently, this decoction has demonstrated promising clinical outcomes in treating CHD (Zhang and Wang, 2017; Lu et al., 2019; Zifeng, 2019; Chen et al., 2020; Jia, 2020; Qi-Min, 2020; Zhang, 2020; Cui et al., 2021; Wang, 2021; Wang et al., 2021; Du et al., 2022; Li, 2022; Hui and Qian, 2023). However, the research conducted to date predominantly features small sample sizes and single-center clinical trials, highlighting a lack of comprehensive, high-quality systematic reviews. Consequently, this study utilizes an evidence-based medicine approach to systematically assess the efficacy of the modified Danggui Sini Decoction alongside conventional Western medicine in treating CHD and angina pectoris, aiming to provide objective, evidence-based recommendations for clinical treatment and drug guidance.

## 2 Methods

This systematic review and meta-analysis were conducted in accordance with the Preferred Reporting Items for Systematic Reviews and Meta-Analyses (PRISMA) Statement (as detailed in Supplementary Material S1). Additionally, this review was registered with PROSPERO (registration number INPLASY202390078).

### 2.1 Literature search

Search terms in Chinese for the modified Danggui Sini Decoction included “Danggui Sini Tang,” “Coronary atherosclerosis,” “Coronary heart disease,” “Coronary atherosclerotic heart disease,” “Angina pectoris,” and “Myocardial ischaemia.” English search terms comprised “modified Danggui Sini Decoction,” “Coronary atherosclerosis,” “Coronary heart disease,” “Myocardial ischemia,” and “Angina pectoris.” These terms were utilized in databases such as the China Knowledge Information Network (CNKI), WanFang Database, VIP Database, Chinese Biomedical Literature Database (CBM), PubMed, Embase, Web of Science. Searches were conducted by title, keywords, subject terms, and free word combinations. The search period extended from the inception of each database up to July 2023. Refer to the supplementary document for a detailed search strategy (Supplementary Table S1).

### 2.2 Inclusion criteria

#### 2.2.1 Literature type

Included were publicly published RCTs both within China and internationally, in both Chinese and English, with no requirement for blinding.

#### 2.2.2 Study subjects

The diagnostic criteria for UA were based on the Guidelines for the Diagnosis and Treatment of Unstable Angina and Non-ST-Segment Elevation Myocardial Infarction, and the Guidelines for the Diagnosis and Treatment of Non-ST-Segment Elevation Acute Coronary Syndromes (2016 Edition) (Lei, 2012; Chinese Medical Association and Division of Cardiovascular Diseases, 2017). Similarly, the diagnosis of Stable Angina (SA) adhered to criteria set forth in the Guidelines for the Diagnosis and Treatment of Chronic Stable Angina (Chinese Medical Association Cardiovascular Disease Branch and Chinese Journal of Cardiovascular Disease Editorial Committee, 2007).

#### 2.2.3 Intervention measures

The control group received conventional Western medicines, while the experimental group was treated with modified Danggui Sini Decoction in addition to the standard treatment.

#### 2.2.4 Outcome measures

The primary efficacy outcomes encompassed the clinical effective rate (including angina pectoris effective rate, electrocardiogram effective rate, nitroglycerin dosage reduction rate, and traditional Chinese medicine syndrome effective rate). Secondary outcomes entailed the traditional Chinese medicine syndrome score [as specified in the “Guidelines for Clinical Research of New Chinese Medicines” (Zheng, 2002)], cardiac function indicators [cardiac output (CO), left ventricular ejection fraction (LVEF), and left ventricular end-diastolic diameter (LVEDD)], angina pectoris episodes (frequency and duration of angina attacks), Seattle Angina Questionnaire (SAQ) score, and inflammatory factors [interleukin-6 (IL-6), tumor necrosis factor-alpha (TNF-α), and high-sensitivity C-reactive protein (hs-CRP)].

### 2.3 Exclusion criteria

Exclusions included duplicate publications, reviews, conference papers, animal studies, case reports, studies with incomplete data or lacking outcome indicators, and studies whose interventions did not align with the inclusion criteria.

### 2.4 Literature screening and data extraction

Two researchers independently conducted literature screening and data extraction based on the inclusion and exclusion criteria. Discrepancies were resolved through discussion or consultation with a third researcher. Extracted data covered authors, publication year, title, sample sizes of test and control groups, demographics (sex, age, disease duration), interventions, treatment duration, and outcome measures.

### 2.5 Quality assessment of included literature

Quality was assessed using the Cochrane Handbook 5.1 “Risk of Bias Assessment” tool, examining six dimensions: random sequence generation, allocation concealment, blinding implementation, data completeness, selective reporting, and other sources of bias. Outcomes were categorized as “low risk,” “high risk,” or “unclear risk” of bias.

### 2.6 Evidence quality evaluation

Use GRADEprofiler 3.6 for evidence quality evaluation, divided into four levels: high (A), medium (B), low (C), and extremely low (D). Whether to downgrade during the evaluation process mainly considers five aspects: research limitations, inconsistency, indirectness, imprecision, and publication bias.

### 2.7 Statistical methods

Meta-analysis was conducted using RevMan 5.4 software. For count data, odds ratios (OR) or risk ratios (RR) were used, while mean difference (MD) or standardized mean difference (SMD) were employed for continuous data, with 95% confidence intervals (CI) calculated. Heterogeneity was assessed by the *I* ^2^ statistic; a fixed-effects model was applied if *p* > 0.10 and *I* ^2^ ≤ 50%, and a random-effects model for *p* ≤ 0.10 and *I* ^2^ > 50%, with subgroup or sensitivity analysis as appropriate. Publication bias was evaluated with a funnel plot for studies including more than 10 outcome measures.

## 3 Results

### 3.1 Results of literature screening

**FIGURE 1:**
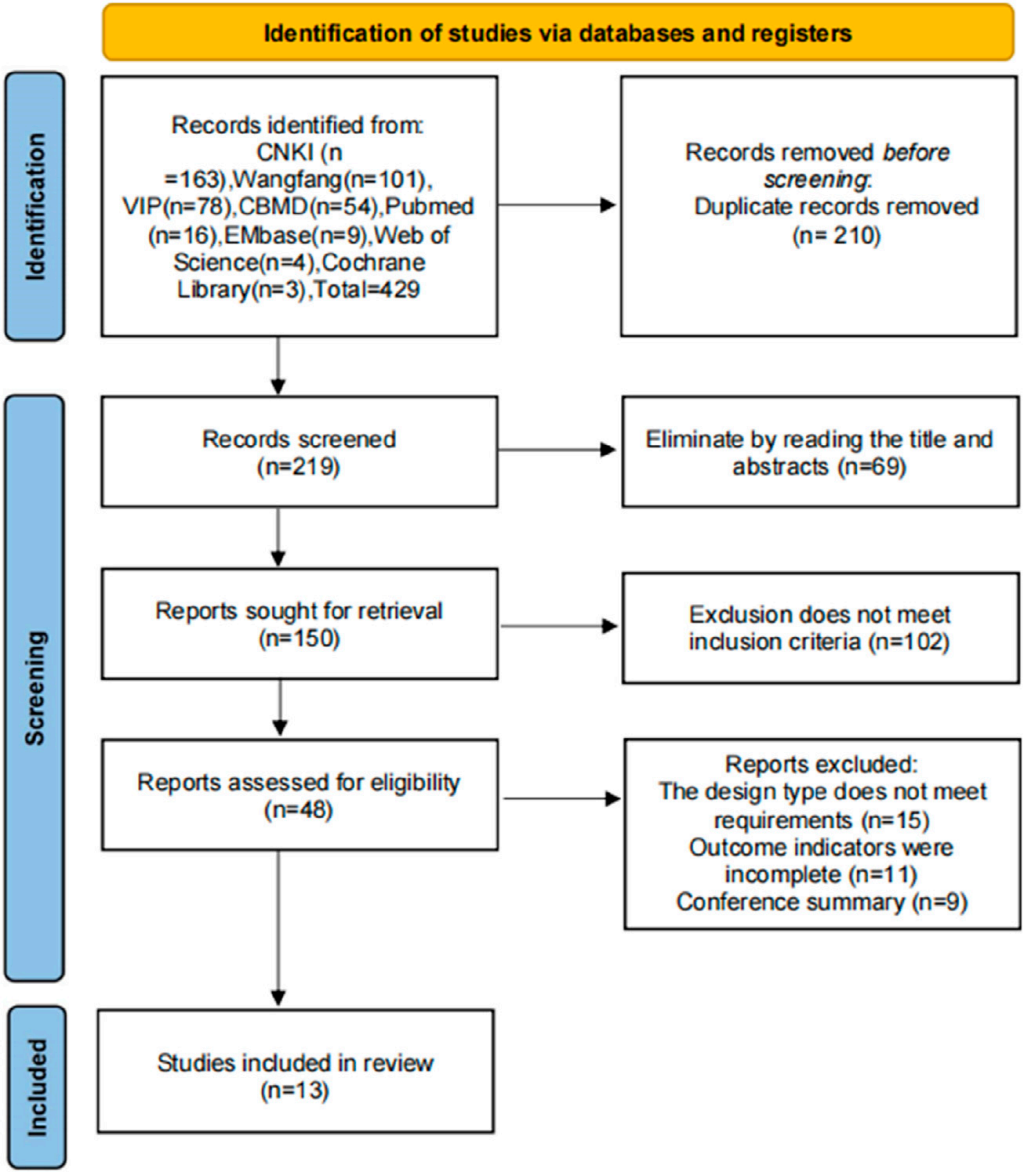
Literature screening process.

A total of 432 pieces of literature were retrieved. After removing duplicates using Endnote software, 219 remained and were assessed according to the inclusion and exclusion criteria, ultimately including 13 studies (Zhang and Wang, 2017; Lu et al., 2019; Zifeng, 2019; Chen et al., 2020; Jia, 2020; Qi-Min, 2020; Zhang, 2020; Cui et al., 2021; Wang, 2021; Wang et al., 2021; Du et al., 2022; Li, 2022; Hui and Qian, 2023). The literature screening process is depicted in Figure 1. In accordance with the “Type A extract” definition from the ConPhyMP consensus statement (Peng et al., 2023), a summary table was compiled to describe the botanical drug components reported in the original studies. As a Type A extract, modified Danggui Sini Decoction comprises six botanical drugs, aimed at warming meridians, dispelling cold, nourishing blood, and unblocking meridians. The core prescription in all studies was modified Danggui Sini Decoction, with additional botanical drugs like Angelica sinensis (Oliv.) Diels (Umbelliferae; *Angelicae sinensis* Radix) (Danggui), *Asarum heterotropoides* F. Schmidt (Aristolochiaceae Juss.) (Xixin), *Cynanchum otophyllum* Schneid. (Contortae.) (Baishao), *Glycyrrhiza uralensis* Fisch. (Fabaceae Lindl.) (Gancao), *Cinnamomum cassia* (L.) D. (DonRamulus Cinnamomi) (Guizhi), *Tetrapanax papyrifer* (Hook.) K. Koch (*Tetrapanax papyriferus*) (Tongcao), adjusted according to syndrome differentiation. The composition of these prescriptions is detailed in Supplementary Table S2.

### 3.2 Basic information of included studies

The review included 13 RCTs, encompassing 1,232 patients—616 in the treatment group and 616 in the control group. Baseline characteristics across studies were comparable, with detailed features presented in Table 1.

**TABLE 1:** Basic characteristics of the included studies.

Subjects	Disease type	Sample size (m/f)		Average age/years		Interventions		Average duration of illness/year		Treatment/week	Outcomes
		T	C	T	C	T	C	T	C		
Hui and Qian (2023)	UA	40(23/17)	40(21/19)	62.17 ± 3.24	61.33 ± 2.97	modified Danggui Sini Decoction+C	CWM	-	-	4	①⑤⑥
Li (2022)	SA	55(30/25)	55(32/23)	67.30 ± 5.71	68.37 ± 7.20	modified Danggui Sini Decoction+C	CWM	2.56 ± 0.58	2.67 ± 0.63	12	①⑤⑦
Du et al., 2022	SA/UA	39(24/15)	39(27/12)	64.87 ± 6.51	65.81 ± 6.24	modified Danggui Sini Decoction+C	CWM	6.14 ± 2.03	6.38 ± 1.53	4	②③④⑤⑧
Cui et al. (2021)	UA	34(13/21)	34(11/24)	55.24 ± 2.21	53.03 ± 1.41	modified Danggui Sini Decoction+C	CWM	4.97 ± 0.21	4.93 ± 0.26	3	①⑤⑦
Wang (2021)	SA	45(26/19)	45(24/21)	70.16 ± 2.32	70.23 ± 2.83	modified Danggui Sini Decoction+C	CWM	5.47 ± 1.31	5.52 ± 1.28	4	①⑤⑥⑦
Wang et al. (2021)	SA/UA	132 (64/68)	132(66/66)	67.61 ± 7.81	66.13 ± 6.92	modified Danggui Sini Decoction+C	CWM	8.15 ± 0.94	8.94 ± 1.12	4	④⑤⑥
Jia (2020)	UA	30(17/13)	30(15/15)	60.23 ± 15.32	61.07 ± 14.8	modified Danggui Sini Decoction+C	CWM	3.01 ± 3.71	3.25 ± 4.2	4	①②③④
Qi-Min (2020)	SA	36(20/16)	36(19/17)	56.23 ± 15.32	58.07 ± 14.8	modified Danggui Sini Decoction+C	CWM	-	-	8	①②③④⑤⑦
Zhang (2020)	SA/UA	48(22/26)	48(25/23)	79.68 ± 9.32	78.07 ± 9.8	modified Danggui Sini Decoction+C	CWM	6.29 ± 2.71	6.04 ± 2.42	4	①⑨⑩
Chen et al. (2020)	UA	30(15/15)	30(18/12)	56.2 ± 3.6	57.4 ± 4.8	modified Danggui Sini Decoction+C	CWM	-	-	4	①⑤⑥⑨
Zifeng (2019)	SA	30(12/18)	30(16/14)	63.23 ± 5.32	64.07 ± 4.8	modified Danggui Sini Decoction+C	CWM	5.3 ± .8	5.1 ± 2.7	6	②③④⑦⑩
Lu et al. (2019)	SA	57(31/26)	57(29/28)	72.16 ± 7.21	73.07 ± 8.02	modified Danggui Sini Decoction+C	CWM	10.11 ± 2.71	9.89 ± 1.94	4	②③④⑤⑥⑧
Zhang and Wang (2017)	SA/UA	40(27/13)	40(15/25)	65.23 ± 7.76	65.15 ± 7.95	modified Danggui Sini Decoction+C	CWM	2.79 ± 1.71	3.25 ± 1.20	12	④⑤⑧

Note: T, trial group; C, control group; −, Not reported; SA, stable angina; UA, unstable angina; CWM, conventional western medicine (including antiplatelet agents, statin lipid-lowering drugs, β-blockers, nitrates, etc.); ①Angina pectoris effective rate; ②Ecg response rate; ③Nitroglycerin reduction and discontinuation rate; ④TCM, symptom Effective rate; ⑤Episodes of angina pectoris (Number of angina attacks, Duration of angina pectoris); ⑥Cardiac function index (CO, LVEF, LVEDD); ⑦TCM, syndrome score; ⑧SAQ, score; ⑨NT-ProBNP; ⑩Inflammatory factors.

### 3.3 Quality assessment of included studies

**FIGURE 2:**
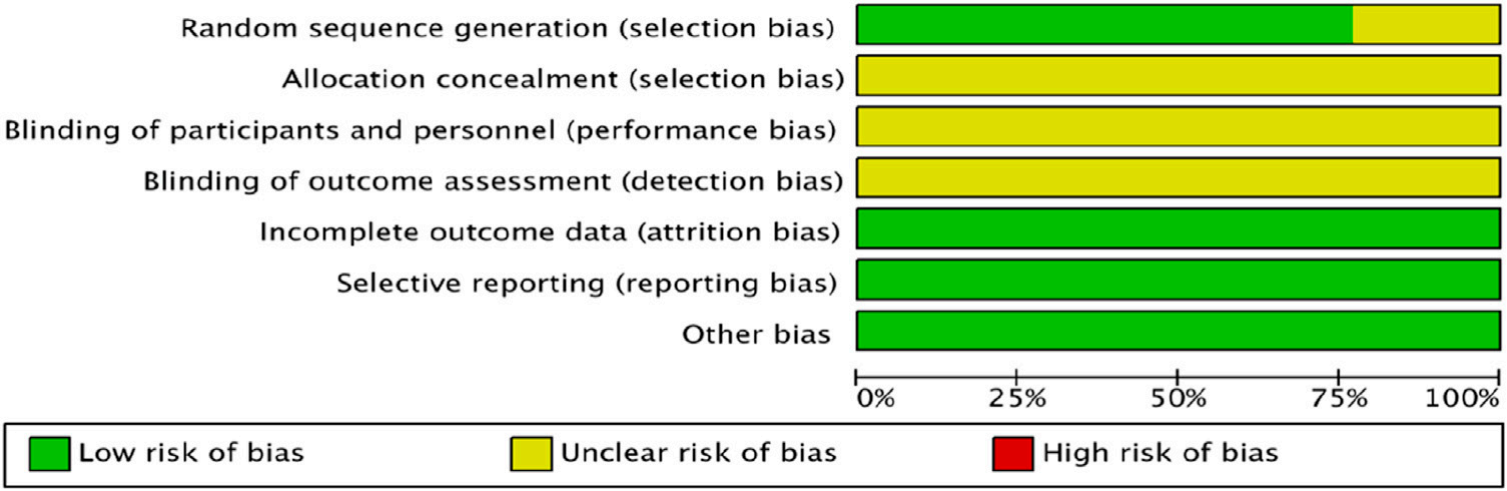
Summary of the risk of bias in the included literature.

**FIGURE 3:**
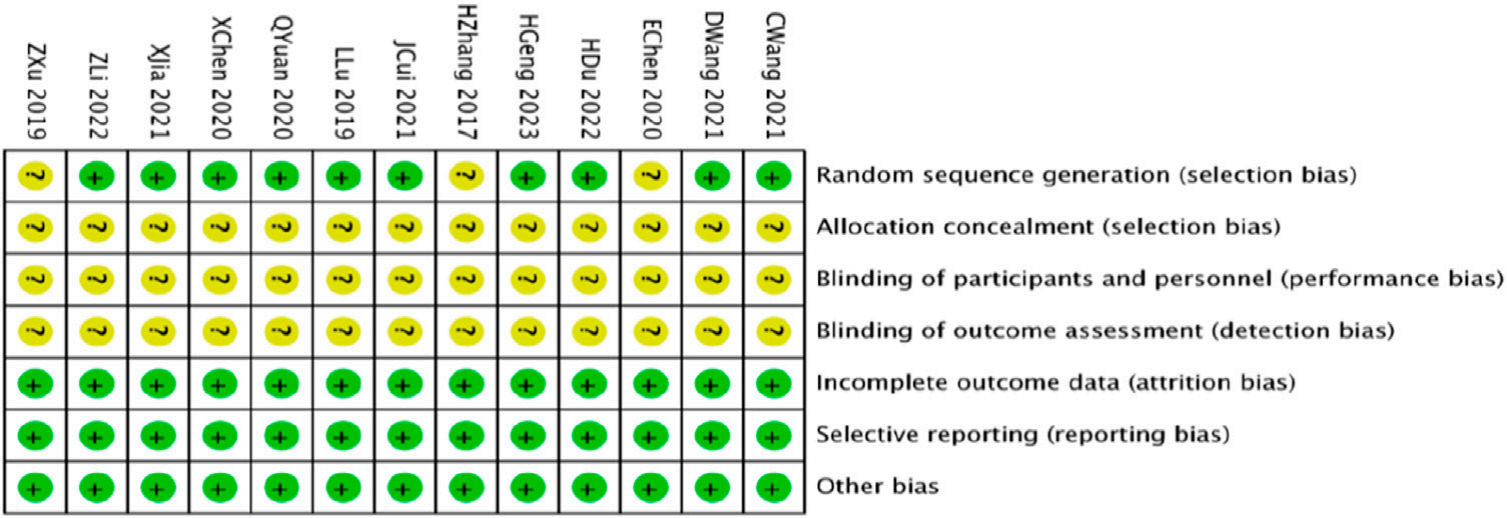
Risk of bias proportional to the risk of inclusion in the literature.

The quality of the studies was assessed using the Cochrane Handbook 5.1 “Risk of Bias Assessment” tool. Ten studies (Lu et al., 2019; Chen et al., 2020; Jia, 2020; Qi-Min, 2020; Cui et al., 2021; Wang, 2021; Wang et al., 2021; Du et al., 2022; Li, 2022; Hui and Qian, 2023) utilized the randomized numeric table method for randomization. Three studies (Zhang and Wang, 2017; Zifeng, 2019; Zhang, 2020) did not clearly specify their randomization techniques; none disclosed details on allocation concealment or blinding, hence were considered at unknown risk. All studies reported complete outcomes, with no evidence of selective outcome reporting or other biases, classifying them at low risk (Figures 2, 3).

### 3.4 Meta-analysis results

#### 3.4.1 Efficacy in angina pectoris

**FIGURE 4:**
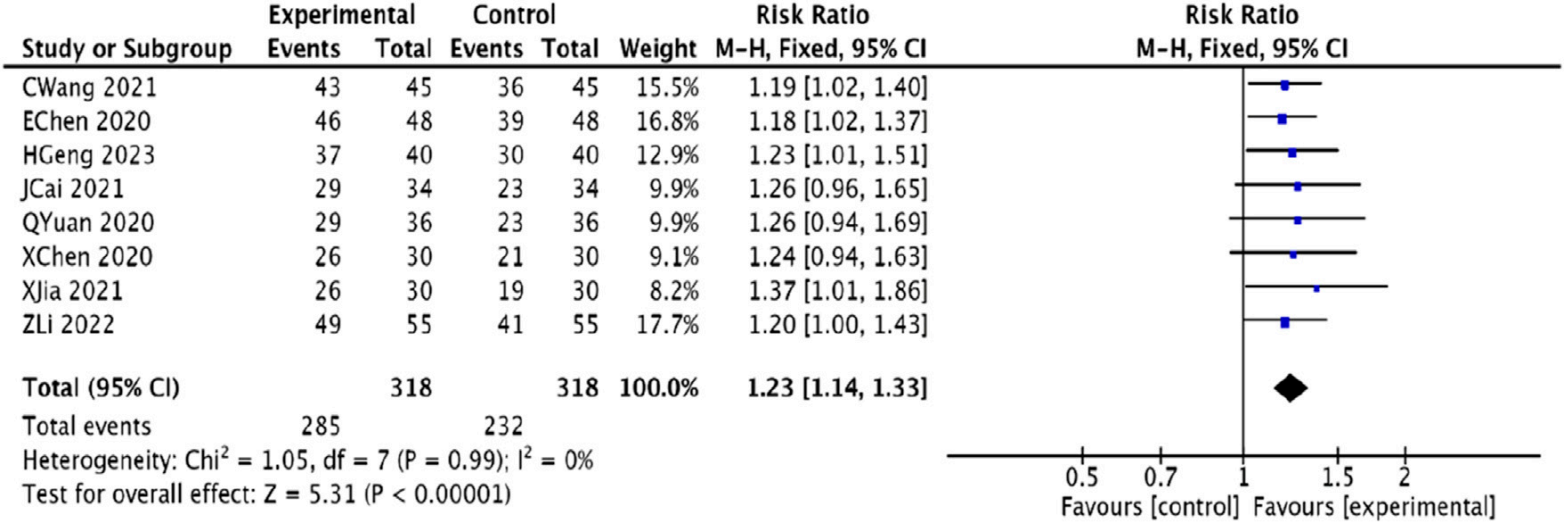
Meta-analysis of the effective rate of angina pectoris in patients with coronary heart disease angina pectoris treated with modified Danggui Sini Decoction in combination with conventional western drugs.

Eight studies (Chen et al., 2020; Jia, 2020; Qi-Min, 2020; Zhang, 2020; Cui et al., 2021; Wang, 2021; Li, 2022; Hui and Qian, 2023) involving 636 patients assessed the efficacy of treatment for angina pectoris. These studies demonstrated homogeneity (*p* = 0.99, *I* ^2^ = 0), and a fixed-effects model was applied for the meta-analysis. The results indicated that the experimental group experienced a significant improvement in the treatment efficacy of angina pectoris compared to the control group, with a statistically significant difference [RR = 1.23, 95% CI (1.14, 1.33), *p* < 0.00001] (Figure 4).

#### 3.4.2 Effective rate of TCM symptoms

**FIGURE 5:**
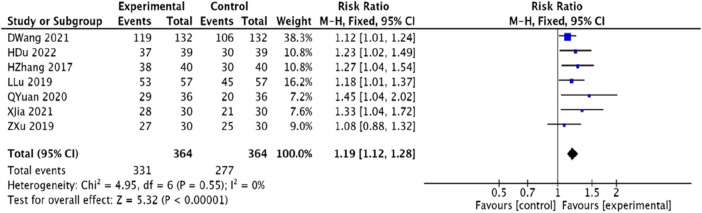
Meta-analysis of the effective rate of Chinese medicine syndromes in patients with coronary heart disease angina pectoris treated with modified Danggui Sini Decoction combined with conventional western medicine.

Seven studies (Zhang and Wang, 2017; Lu et al., 2019; Zifeng, 2019; Jia, 2020; Qi-Min, 2020; Wang et al., 2021; Du et al., 2022), totaling 728 patients, reported on the effective rate of TCM symptoms. The analysis found homogeneity among the studies (*p* = 0.55, *I* ^2^ = 0), and a fixed-effects model was employed. The findings revealed that, compared to the control group, the experimental group significantly improved the TCM symptomatic effective rate, with a statistically significant difference [RR = 1.19, 95% CI (1.12, 1.28), *p* < 0.000 01].(Figure 5).

#### 3.4.3 Nitroglycerin usage reduction

**FIGURE 6:**
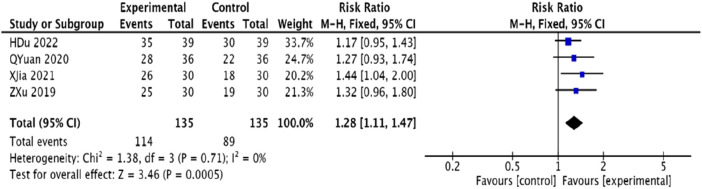
Meta-analysis of the rate of nitroglycerin reduction in patients with coronary heart disease angina pectoris treated with modified Danggui Sini Decoction in combination with conventional western medicines.

Four studies (Lu et al., 2019; Zifeng, 2019; Qi-Min, 2020; Du et al., 2022), comprising 270 patients, examined the reduction in nitroglycerin usage. These studies showed homogeneity (*p* = 0.71, *I* ^2^ = 0), allowing for a fixed-effects model in the meta-analysis. The outcomes demonstrated that the experimental group significantly reduced nitroglycerin usage compared to the control group, with a statistically significant difference [RR = 1.28, 95% CI (1.11, 1.47), *p* = 0.0005]. Refer to Figure 6.

#### 3.4.4 ECG effectiveness rate

**FIGURE 7:**
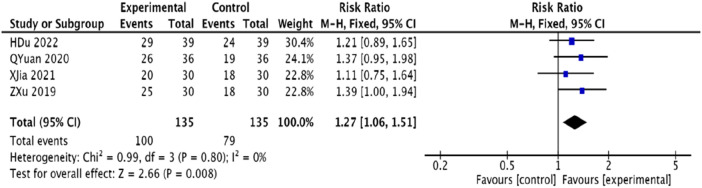
Meta-analysis of the ECG efficiency of modified Danggui Sini Decoction combined with conventional western drugs in the treatment of patients with angina pectoris in coronary artery disease.

The effectiveness of ECG improvements was reported in four studies (Lu et al., 2019; Zifeng, 2019; Qi-Min, 2020; Du et al., 2022) with a total of 270 patients. The analysis showed homogeneity (*p* = 0.80, *I* ^2^ = 0), and a fixed-effects model was utilized. Results indicated that the trial group significantly improved ECG efficiency in patients compared to the control group, with a statistically significant difference [RR = 1.27, 95% CI (1.06, 1.51), *p* = 0.008] (Figure 7).

#### 3.4.5 Number of angina attacks

**FIGURE 8:**
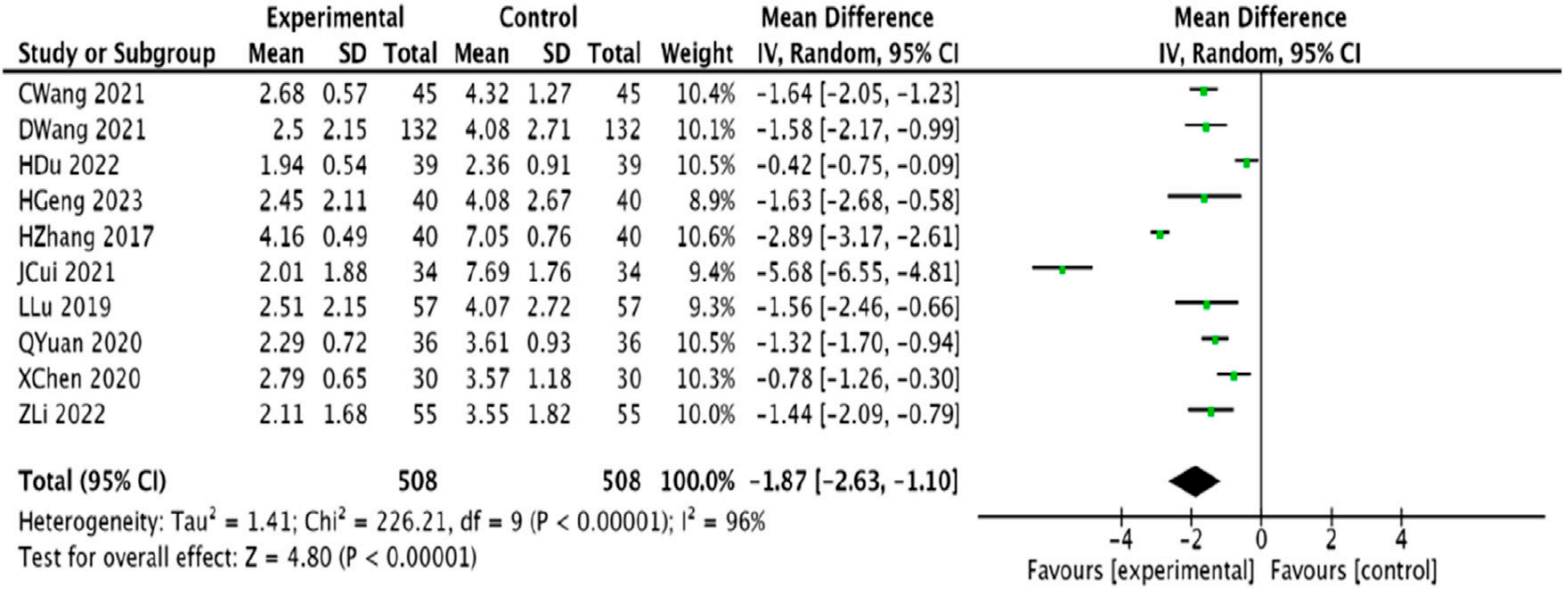
Meta-analysis of the combination of modified Danggui Sini Decoction with conventional western drugs to reduce the number of angina pectoris in patients with coronary heart disease angina pectoris.

Ten studies (Zhang and Wang, 2017; Lu et al., 2019; Chen et al., 2020; Qi-Min, 2020; Cui et al., 2021; Wang, 2021; Wang et al., 2021; Du et al., 2022; Li, 2022; Hui and Qian, 2023), involving 1,016 patients, reported on the number of angina episodes. There was significant heterogeneity among the studies (*p* < 0.00001, *I* ^2^ = 96%); thus, a random-effects model was adopted, which revealed that the trial group effectively reduced the number of angina episodes compared with the control group, with a statistically significant difference (MD = −1.87, 95% CI [−2.63, −1.10], *p* < 0.00001) (Figure 8).

#### 3.4.6 Duration of angina pectoris

**FIGURE 9:**
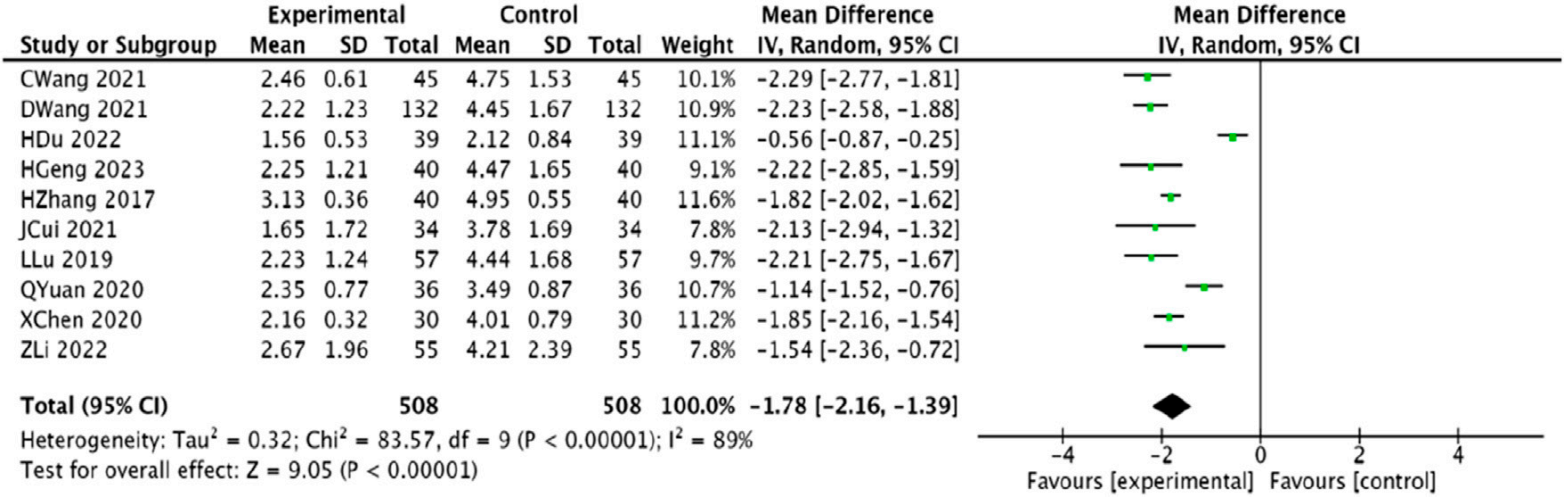
Meta-analysis of the combination of modified Danggui Sini Decoction with conventional western drugs to shorten the duration of angina in patients with coronary angina pectoris.

Ten studies (Zhang and Wang, 2017; Lu et al., 2019; Chen et al., 2020; Qi-Min, 2020; Cui et al., 2021; Wang, 2021; Wang et al., 2021; Du et al., 2022; Li, 2022; Hui and Qian, 2023), encompassing 1,016 patients, reported on the duration of angina pectoris. Significant heterogeneity was observed among the studies (*p* < 0.00001, *I* ^2^ = 89%), prompting the use of a random-effects model. The meta-analysis revealed that the trial group significantly reduced the duration of angina pectoris compared to the control group, with a statistically significant difference [MD = −1.78, 95% CI (−2.16, −1.39), *p* < 0.00001] (Figure 9).

#### 3.4.7 Indicators of cardiac function

**FIGURE 10:**
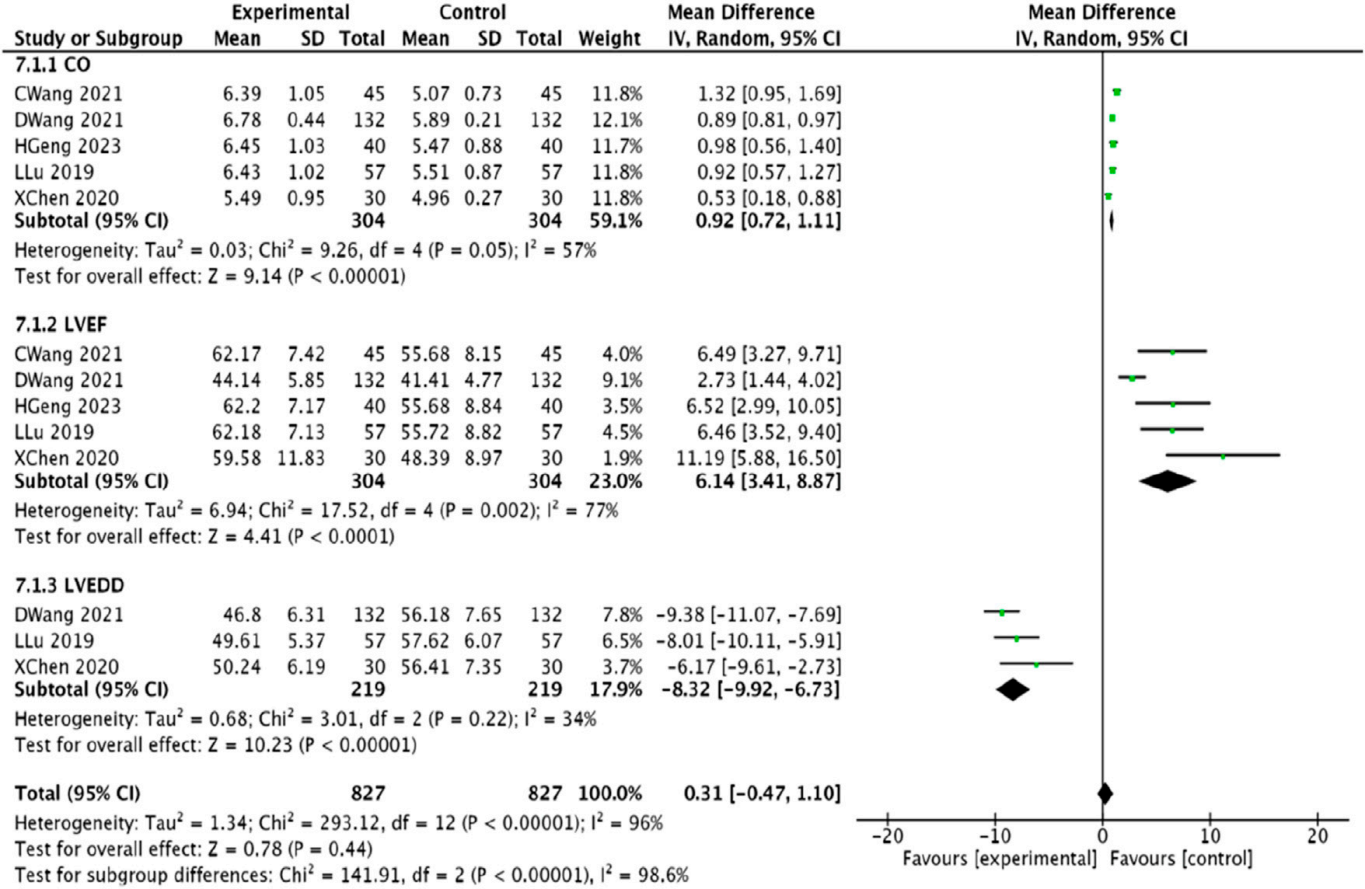
Meta-analysis of modified Danggui Sini Decoction combined with conventional western drugs to improve cardiac function in patients with coronary angina pectoris.

Five studies (Lu et al., 2019; Chen et al., 2020; Wang, 2021; Wang et al., 2021; Hui and Qian, 2023) evaluated CO and LVEF indices, while three studies (Chen et al., 2020; Wang et al., 2021; Hui and Qian, 2023) examined LVEDD. Due to heterogeneity among the findings, a random-effects model was employed for the meta-analysis. The results indicated improvements in CO [MD = 0.92, 95% CI (0.72, 1.11), *p* < 0.00001], LVEF [MD = 6.14, 95% CI (3.41, 8.87), *p* < 0.0001], and LVEDD [MD = −8.32, 95% CI (−9.92, −6.73), *p* < 0.00001], demonstrating that the experimental group significantly enhanced cardiac function compared to the control group (Figure 10).

#### 3.4.8 TCM symptom score

**FIGURE 11:**
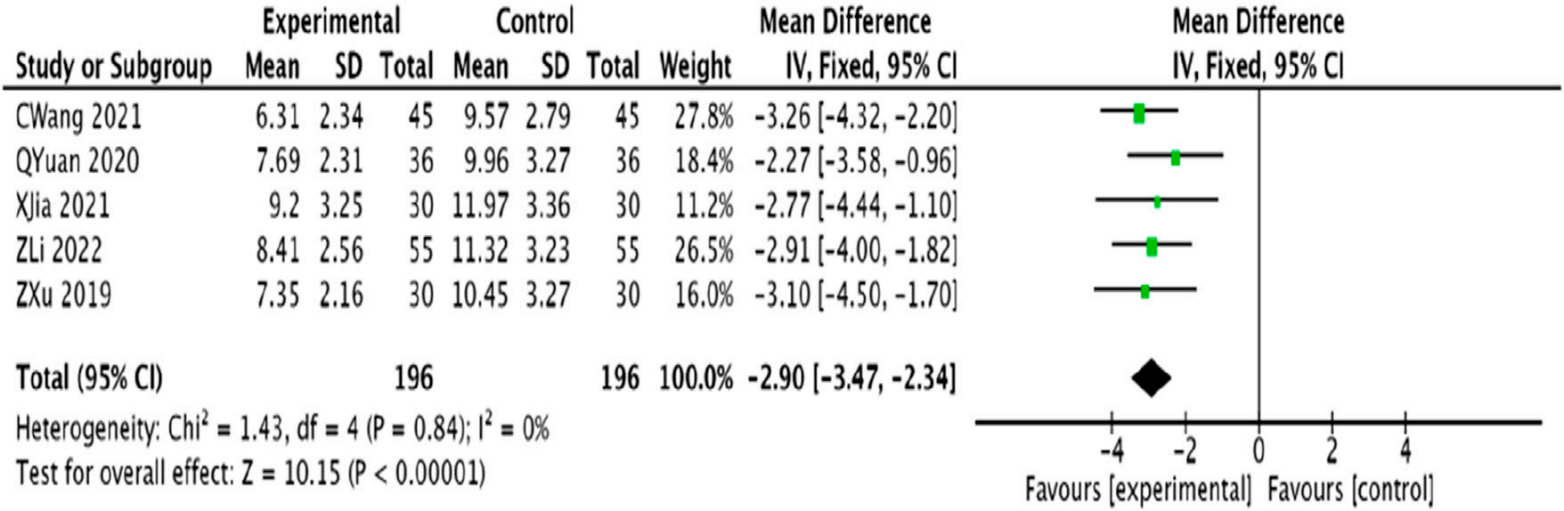
Meta-analysis of the improvement of TCM symptom scores in patients with angina pectoris of coronary artery disease by combining modified Danggui Sini Decoction with conventional western drugs.

Five studies (Zifeng, 2019; Jia, 2020; Cui et al., 2021; Wang, 2021; Li, 2022), involving 392 patients, assessed TCM symptom scores. The studies showed homogeneity (*p* = 0.84, *I* ^2^ = 0), leading to the application of a fixed-effect model. The analysis showed that the experimental group significantly reduced TCM syndromic scores compared to the control group, with a statistically significant difference [MD = −2.90, 95% CI (−3.47, −2.34), *p* < 0.00001] (Figure 11).

#### 3.4.9 Seattle angina questionnaire

**FIGURE 12:**
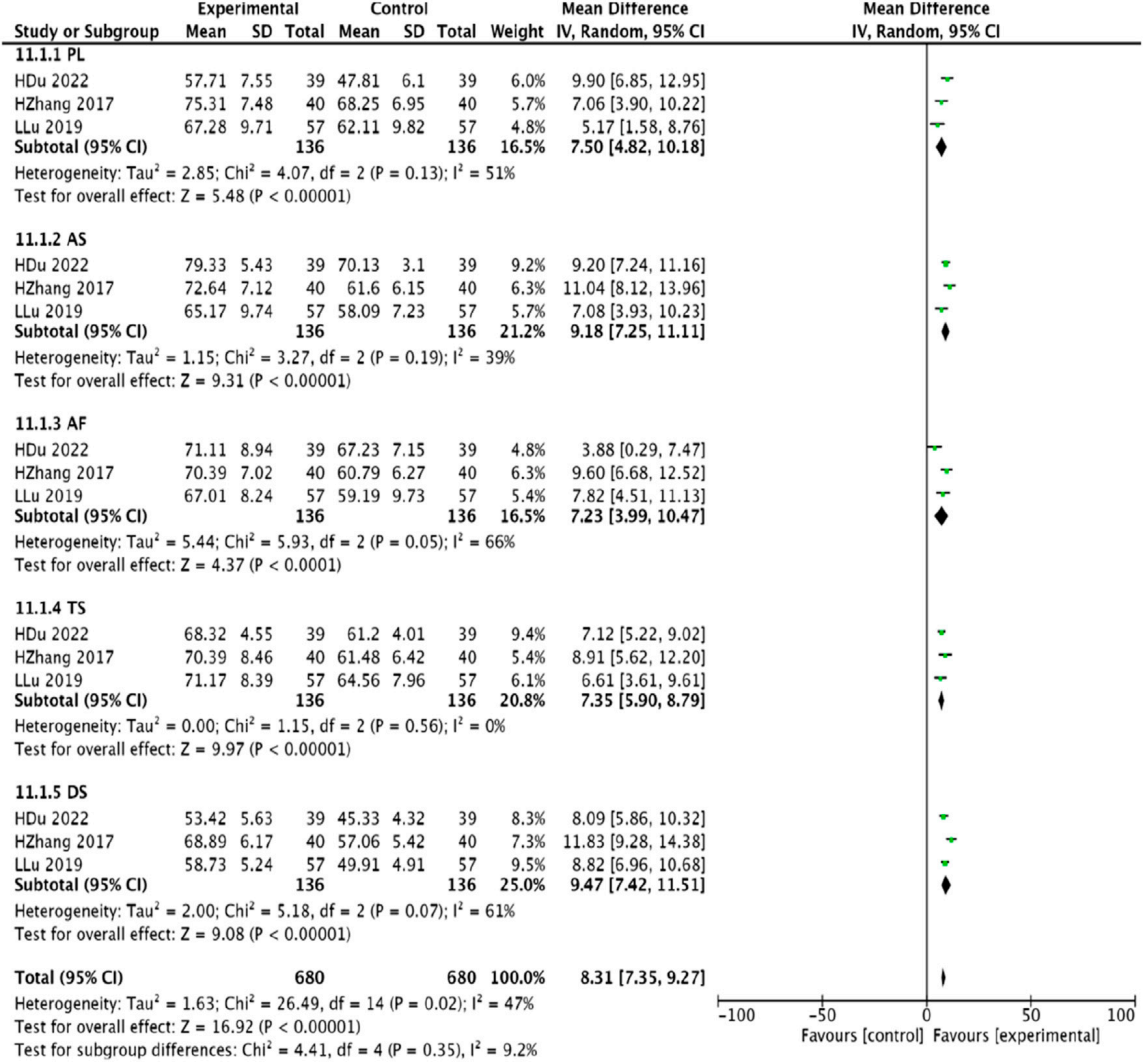
Meta-analysis of the improvement of SAQ scores in patients with coronary angina pectoris by combining modified Danggui Sini Decoction with conventional western drugs.

Three studies (Zhang and Wang, 2017; Lu et al., 2019; Du et al., 2022), comprising 272 patients, referenced the Seattle Angina Questionnaire. Given the homogeneity of these studies, a random-effects model was conducted for meta-analysis. The findings highlighted significant improvements in the limitation of physical activity [MD = 7.50, 95% CI (4.82, 10.18), *p* < 0.00001], stability of angina [MD = 9.18, 95% CI (7.25, 11.11), *p* < 0.00001], frequency of anginal attacks [MD = 7.23, 95% CI (3.99, 10.47), *p* < 0.00001], treatment satisfaction [MD = 7.35, 95% CI (5.90, 8.79), *p* < 0.0001], and disease awareness (MD = 9.47, 95% CI (7.42, 11.51), *p* < 0.00001), demonstrating the experimental group’s effectiveness in improving patient symptoms of angina compared to the control group (Figure 12).

#### 3.4.10 NT-proBNP

**FIGURE 13:**
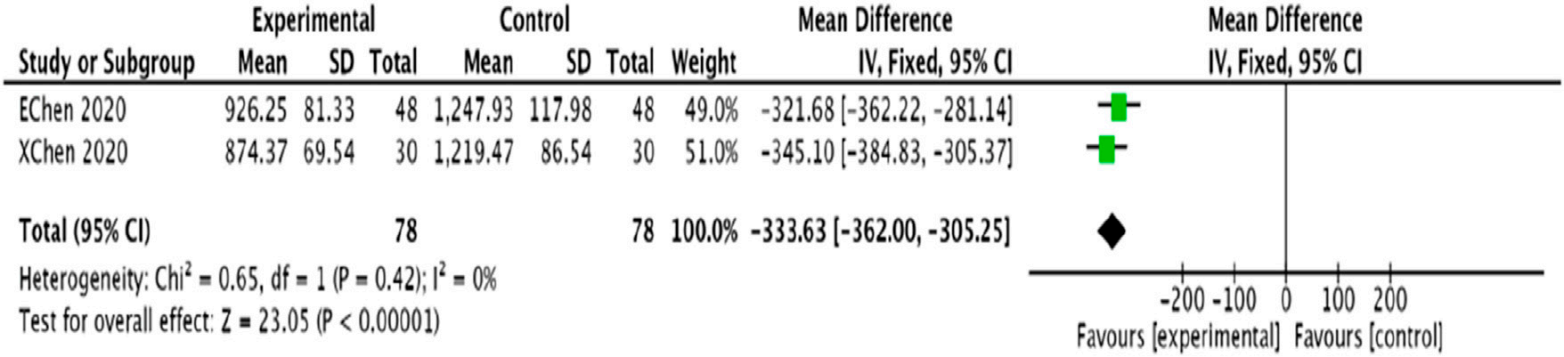
Meta-analysis of modified Danggui Sini Decoction combined with conventional western drugs to improve NT-ProBNP in patients with coronary angina pectoris.

Two studies (Chen et al., 2020; Zhang, 2020) assessed NT-proBNP levels in 156 patients. Given the homogeneity observed among the studies (*p* = 0.42, = 0), a fixed-effects model was employed for the meta-analysis. The results demonstrated that, compared to the control group, the experimental group significantly reduced NT-ProBNP levels, with a statistically significant difference [MD = −333.63, 95% CI (−362.00, −305.25), *p* < 0.00001] (Figure 13).

#### 3.4.11 Inflammatory factors

**FIGURE 14:**
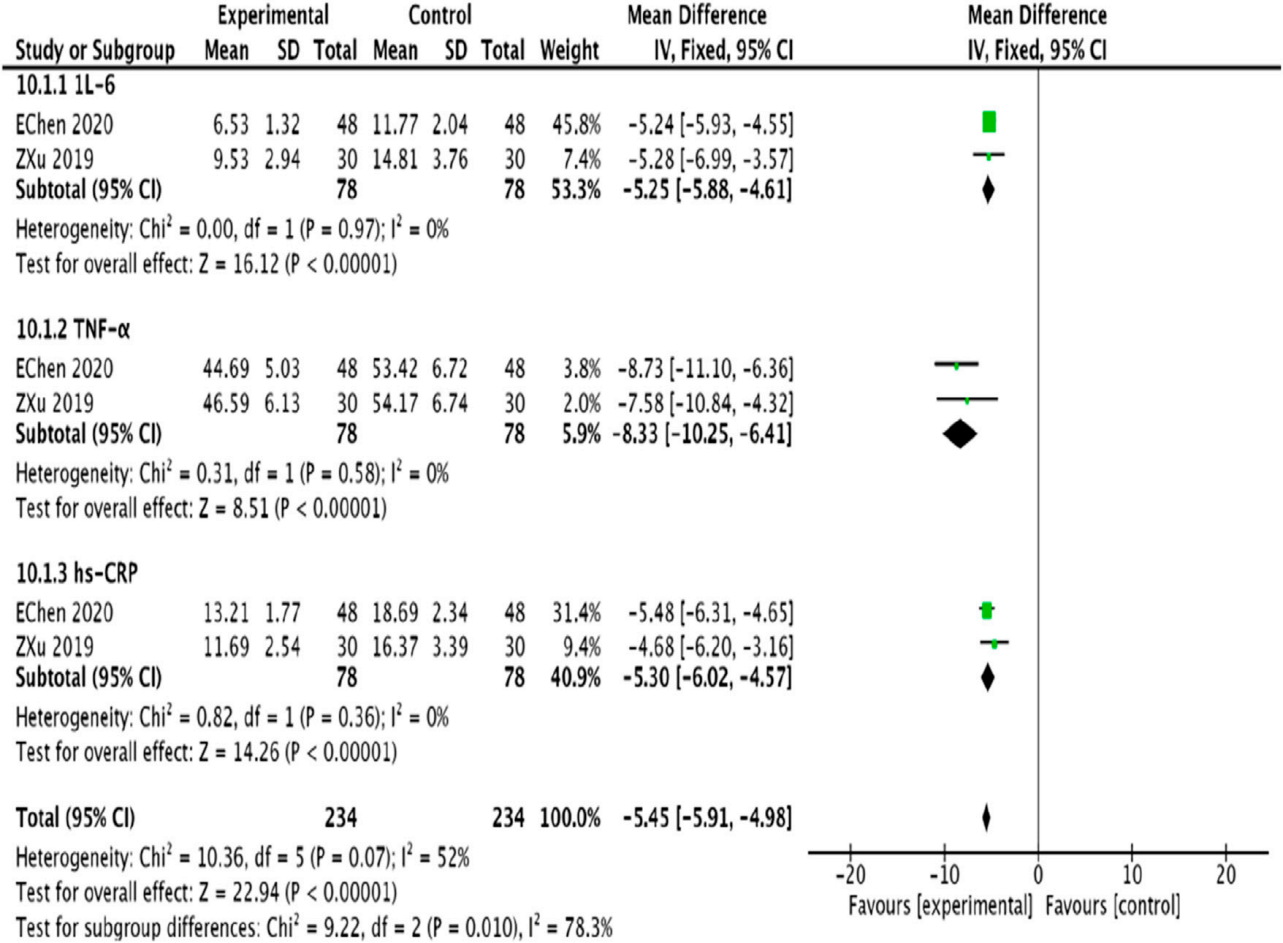
Meta-analysis of the improvement of inflammatory factors in patients with coronary heart disease angina pectoris by combining modified Danggui Sini Decoction with conventional western medicines.

Two studies (Zifeng, 2019; Zhang, 2020) examined IL-6, TNF-α, and hs-CRP levels. After testing for homogeneity, a fixed-effects model was applied for the meta-analysis. The findings indicated significant reductions in IL-6 [MD = −5.25, 95% CI (−5.88, −4.61), *p* < 0.00001], TNF–α (MD = −8.33, 95% CI (−10.25, −6.41), *p* < 0.00001), and hs-CRP [MD = −5.30, 95% CI (−6.02, −4.57), *p* < 0.00001] in the experimental group compared to the control group, effectively reducing inflammatory markers (Figure 14).

#### 3.4.12 Adverse events rates

**FIGURE 15:**
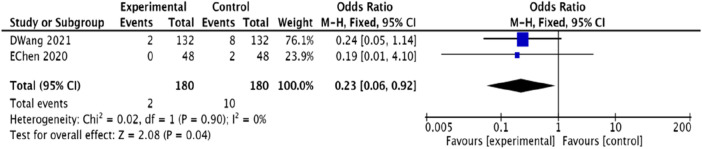
Meta-analysis of the improvement of adverse events rates in patients with coronary heart disease angina pectoris by combining modified Danggui Sini Decoction with conventional western medicines.

Two studies (Zhang, 2020; Wang et al., 2021) reported on adverse events, noting that two patients in the experimental group experienced thirst, while 10 patients in the control group suffered from decreased appetite, diarrhea, and gastrointestinal symptoms. The homogeneity test showed consistency among the studies. The meta-analysis results [OR = 0.23, 95% CI (0.06, 0.92), *p* = 0.04] indicated that the experimental group had a better safety profile than the control group. These results are depicted in Figure 15.

#### 3.4.13 Risk of publication bias assessment

**FIGURE 16:**
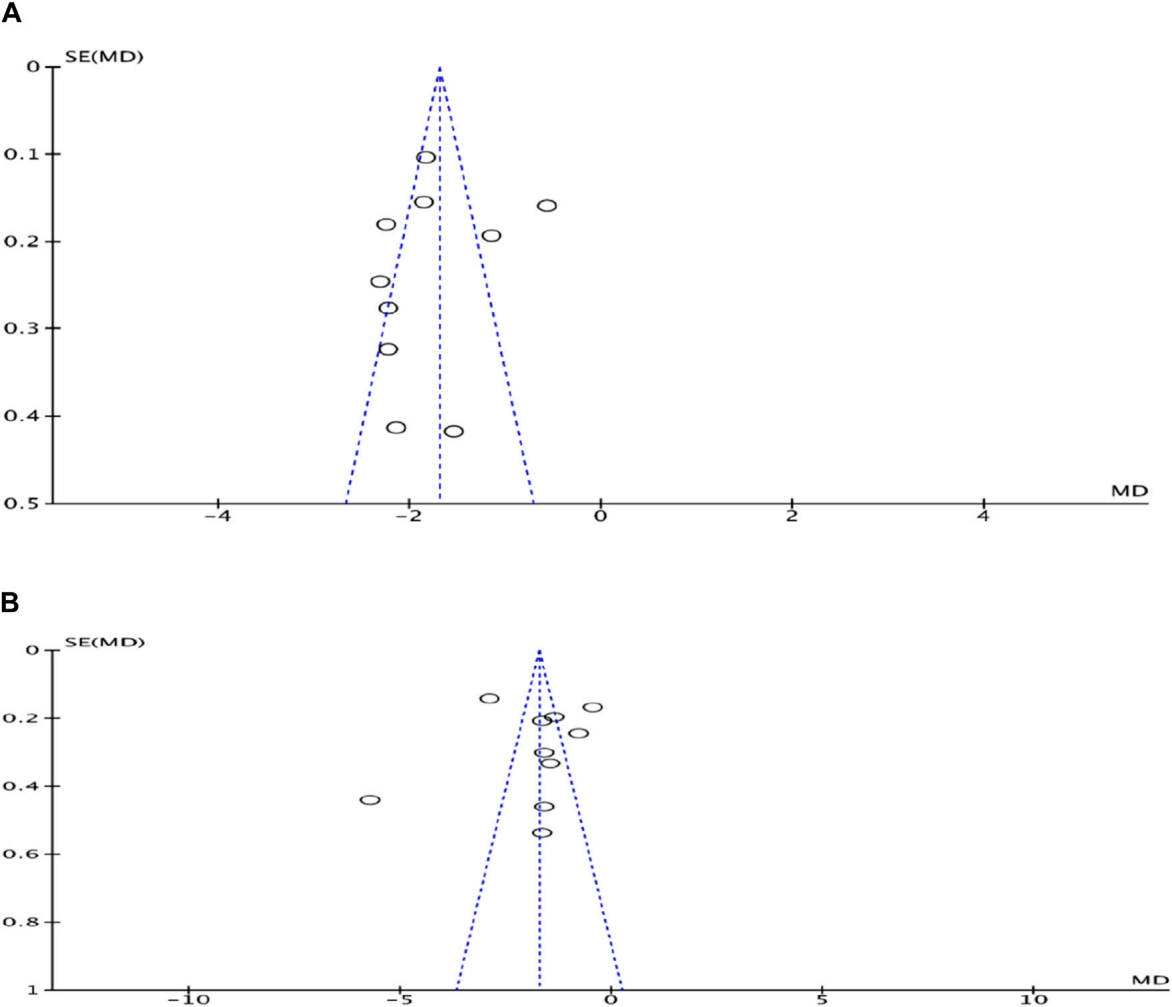
**(A)** Funnel plot of the number of episodes of angina pectoris in coronary artery disease treated with modified Danggui Sini Decoction combined with conventional western drugs. **(B)** Funnel plot of duration of angina pectoris in coronary artery disease treated with modified Danggui Sini Decoction in combination with conventional western drugs.

A funnel plot was used to evaluate the risk of publication bias for the outcome indicators concerning the number of angina episodes and the duration of angina. The left-right asymmetry observed in the plot of each study point suggests a potential for publication bias (Figures 16A,B).

### 3.5 Evidence quality evaluation

The GRADE evaluation results of the evidence of Danggui Sini Tang in assisting the treatment of coronary heart disease angina pectoris patients can be found in Supplementary Material S2. The main reasons for the downgrading of bias risk include bias caused by missing blinding and insufficient allocation concealment in the included studies; The main reason for the inconsistency degradation is that there is significant heterogeneity among some studies without reasonable explanations, which may affect the scientific validity of research methods and the reliability of research results; The main reason for the degradation of imprecision is that the confidence interval is too large, which affects the accuracy of the evidence.

## 4 Discussion

According to the China Cardiovascular Health and Disease Report 2019, cardiovascular disease affects approximately 330 million individuals in China, with the burden of disease on the rise (Layang, 2016). Among these, around 11 million are currently suffering from CHD, highlighting the growing focus on the prevention and treatment of CHD. Angina pectoris, a significant subtype of CHD, is noted for its high prevalence, mortality rate, treatment costs, and generally poor prognosis (Wang et al., 2023). Consequently, there is an increasing interest in traditional medicines, with the integration of Chinese and Western medicine in CHD treatment emerging as a 21st-century focal point. This approach has shown substantial progress in enhancing physical activity tolerance and symptom relief and is gaining acceptance among healthcare professionals and patients (Yafang et al., 2019). Thus, exploring and understanding the trends and patterns in the combined use of traditional Chinese and Western medicines for CHD treatment is essential.

Coronary artery disease, resulting from coronary atherosclerosis or thrombosis, leads to myocardial ischemia and hypoxia due to the narrowing or blockage of the artery lumen. The inflammatory response and abnormal platelet activation are pivotal in thrombosis, forming the pathological basis of coronary artery thrombosis (Ruddox et al., 2017). Percutaneous coronary intervention (PCI), a primary treatment, does not alter the underlying pathology, hence the risk of in-stent thrombosis and coronary in-stent restenosis (ISR) remains, potentially causing adverse cardiovascular events (Wan, 2023). Post-PCI, antiplatelet therapy is essential, with dual antiplatelet therapy comprising aspirin and clopidogrel recommended for managing coronary artery disease. Clopidogrel, an adenosine diphosphate (ADP) receptor antagonist, together with aspirin, which does not inhibit ADP-induced platelet aggregation alone, can prevent thrombin and platelet activation when combined. Nonetheless, clopidogrel resistance often necessitates increased dosages, the addition of a third antiplatelet drug (aspirin, clopidogrel, cilostazol), P2Y12 receptor antagonists, or the incorporation of TCM (Liu et al., 2013; Yu and Wang, 2014; Zhou and Wang, 2014). Notably, the China Food and Drug Administration has endorsed over 200 proprietary Chinese medicines for adjunctive or complementary angina pectoris treatments, significantly contributing to the reduction of primary end-stage events, anginal episodes, and improvement in electrocardiograms (Quan et al., 2004).

Modified Danggui Sini Decoction, originally detailed in Zhang Zhongjing’s “Shang Han Lun,” comprises Angelicae Sinensis Radix, Ramulus Cinnamomi, Asarum sieboldii Miq, Tetrapanax papyriferus, and Paeonia lactiflora Pall. It is recognized for its ability to warm Yang, activate the veins, promote blood circulation, and eliminate blood stasis (Xiong et al., 2015). Modern research has identified that Angelica sinensis contains diverse components such as volatile oils, terpenes, organic acids, polysaccharides, flavonoids, alkaloids, trace elements, and amino acids (Yi et al., 2005; Yi et al., 2007a; Yi et al., 2007b). Recent studies have isolated “ferulic acid” from Angelica sinensis, highlighting its vascular protective properties and capacity to inhibit platelet aggregation (Li et al., 2006). Additionally, the volatile oil from Angelica sinensis has been found effective in alleviating vasospasm and expanding blood vessels (Quan et al., 2004). Cinnamaldehyde, derived from Cinnamomum cassia, inhibits collagen- and thrombin-induced platelet aggregation both *in vitro* and *in vivo* (Huang et al., 2006). Compounds such as caffeic acid, isochlorogenic acid C, chlorogenic acid, and wild baicalin from Cynanchum officinale exhibit vasodilatory and smooth muscle relaxing properties, besides inhibiting thrombosis *in vivo* (CHEN et al., 2010). The primary active constituents of Paeonia lactiflora, mainly terpenes and terpene glycosides, act on vascular endothelium to dilate vascular smooth muscle, enhancing myocardial blood flow and oxygen and blood supply (Yan et al., 2023). Pharmacological studies have demonstrated that extracts from Tongzhi exhibit significant antithrombotic and anti-inflammatory activities, effectively preventing thrombus formation (Xue et al., 2019). Ginger has been shown to improve blood lipids, facilitate cholesterol excretion, reduce arteriosclerosis (AS) progression, and inhibit platelet aggregation through the activation of signaling pathways such as PI3K-Akt, IL-17, HIF-1, and p53, and the regulation of genes like TP53, MAPK3, MAPK1, AKT1, ESR1, and JUN, thereby mitigating hypoxia-induced cardiac muscle injury and aiding in the management of angina pectoris (Jiang et al., 2023). Various studies confirm that Angelica Siwei Tang possesses anticoagulant and antithrombotic effects, enhances vascular perfusion, and offers anti-inflammatory and analgesic benefits, thereby protecting cardiomyocytes and improving clinical outcomes.

This study was informed by the integration of Chinese and Western medicinal theories, adopting an evidence-based approach to demonstrate that modified Danggui Sini Decoction, in conjunction with conventional Western medications, can effectively enhance clinical outcomes for patients with coronary heart disease coexisting with angina pectoris. This includes improvements in inflammatory markers, cardiac function, and frequency of angina episodes, thereby contributing to disease progression control. Nonetheless, this study is subject to certain limitations. Firstly, among the 13 included RCTs, 3 (Zhang and Wang, 2017; Zifeng, 2019; Zhang, 2020) did not clearly detail their randomization methods; none of the studies disclosed the concealment of allocation schemes or the application of blinding. Secondly, the dosage and treatment duration of modified Danggui Sini Decoction varied across the studies. Thirdly, the geographical location of all included RCTs being in China introduces potential geographic bias, all of which could influence the outcomes. These conclusions warrant confirmation through high-quality research.

## 5 Conclusion

Drawing from the existing evidence, fthe combination of modified Danggui Sini Decoction and conventional Western medicines significantly enhances clinical efficiency, cardiac function, and the management of angina episodes and inflammatory markers, alongside notably improving SAQ scores compared to control treatments, thus offering increased safety. This provides substantiated evidence for its adjunctive use in treating this condition. However, due to several limitations, these findings require further validation through more rigorous clinical studies and foundational research in the future.

## Data Availability

The raw data supporting the conclusions of this article will be made available by the authors, without undue reservation.
