# Supplementary material for: Effects of modified Danggui Sini Decoction as adjuvant therapy for angina pectoris in coronary heart disease: a systematic review and meta-analysis based on randomised controlled trials
Source: Front Pharmacol. 2024 Jun 4;15:1375795. doi: 10.3389/fphar.2024.1375795 (PMC11183329; doi:10.3389/fphar.2024.1375795)
Supplement: Supplementary file 1 [file DataSheet1.pdf]

Supplementary Material

Supplementary Table S1 Search strategy 2

Supplementary Table S2 The composition of the prescriptions 5

Supplementary File S1 The PRISMA checklist of this meta-analysis 20

Supplementary File S2 GRADE quality of evidence 24

Supplementary Table S1 Search strategy

Databases	Search items	Number
**PubMed**	#l:"angina pectoris" [MeSH Terms] OR"coronary heart disease" [Title/ Abstract]OR "Atherosclerosis"[Title/Abstract]OR"Coronary atherosclerosis" [Title/Abstract]<br><br>#2:"Danggui Sini Decoction" [MeSH Terms] OR"Danggui Sini Tang" [Title/ Abstract]OR "Danggui Sini " [Title/Abstract]<br><br>#3:"randomized controlled trial"[Publication Type] OR "controlled clinical trial"[Publication Type] OR "randomized"[Title/Abstract] OR "placebo"[Title/Abstract] OR "randomly"[Title/Abstract] OR "trial"[Title/Abstract]OR "groups"[Title/Abstract]<br><br>#4:#1 AND #2 AND #3	16
**Cochrane Library**	#1 MeSH descriptor: [angina pectoris] explode all trees<br><br>#2(coronary heart disease):ti,ab,kw OR (Coronary atherosclerosis):ti,ab,kw OR (Atherosclerosis):ti,ab,kw<br><br>#3 #1 OR #2<br><br>#4 MeSH descriptor: [Danggui Sini Decoction] explode all trees<br><br>#5 (Danggui Sini Tang):ti,ab,kw OR (Danggui Sini):ti,ab,kw<br><br>#6 #4 or #5<br><br>#7 #3 and #6	3
**Web of science**	#1TS=(angina pectoris OR"coronary heart disease*"OR"Coronary atherosclerosis*"OR"Atherosclerosis*")	4

	#2TS=("Danggui Sini Decoction "OR"Danggui Sini"OR"Danggui Sini Tang")<br><br>#3 #1 AND #2<br><br>#4TS=("randomized controlled trial" OR"controlled clinical<br>trial" OR "randomized*" OR "placebo*" OR"randomly " OR "trial*")<br><br>#5 #3 AND #4	
**Embase**	#1 'angina pectoris'/exp<br>#2 'coronary heart disease':ab,ti<br>#3 'gout suppressants':ab,ti<br>#4 'Coronary atherosclerosis':ab,ti<br>#5 #1 OR #2 OR #3 OR #4<br>#6 'Danggui Sini decoction'/exp<br>#7 'Danggui Sini tang':ab,ti<br>#8 'Danggui Sini':ab,ti<br>#9 #6 OR #7 OR #8<br>#10 'randomized controlled trial'/exp<br>#11 'controlled clinical trial': ti,ab OR 'randomized*':ti,ab OR 'placebo*':ti,ab OR 'randomly':ti,ab OR 'trial*': ti,ab<br>#12 #10 OR #11<br>#10 #5AND #9 AND #12	9
**CNKI**	(SU='冠心病'+'心绞痛'+'冠状动脉粥样硬化型心脏病') AND (SU='当归四逆汤')	163
**Wanfang Data**	(主题:("冠心病" or "心绞痛" or "冠状动脉粥样硬化型心脏病") or 题名或关键词:("冠心病" or "心绞痛" or "冠状动脉粥样硬化型心脏病") or 摘要:("冠心病" or "心绞痛" or "冠状动脉粥样硬化型心脏病")) and (主题:("当归四逆汤") or 题名或关键词:("当归四逆汤") or 摘要:("当归四逆汤"))	101
**VIP**	((M=冠心病 OR 心绞痛 OR 冠状动脉粥样硬化型心脏病) OR (K=冠心病 OR 心绞痛 OR 冠状动脉粥样硬化型心脏病) OR (R=冠心病 OR 心绞痛 OR 冠状动脉粥样硬化型心脏病)) AND ((M=当归四逆汤) OR (K=当归四逆汤) OR (R=当归四逆汤))	78

**CBM**	("冠心病"[摘要:智能] OR "心绞痛"[摘要:智能] OR "冠状动脉粥样硬化型心脏病"[摘要:智能]) AND ("当归四逆汤"[摘要:智能])	54

Supplementary Table S2 The composition of the prescriptions

Study	Chinese name	Species, concentration	Quality control reported? (Y/N)	Chemical analysis reported? (Y/N)
HGeng 2023[12]	Danggui,10g	Angelicae Sinensis Radix [Umbelliferae](Danggui),10g	N	N
	Xixin,10g	Asarum heterotropoides F. Schmidt [Aristolochiaceae Juss.],10g		
	Baishao,10g	Cynanchum otophyllum Schneid.[Contortae.],10g		
	Gancao,8g	Glycyrrhiza uralensis Fisch.[Fabaceae Lindl.],8g		
	Guizhi,10g	Cinnamomum cassia (L.) D.[DonRamulus Cinnamomi],10g		

	Tongcao,12g	Tetrapanax papyrifer (Hook.) K. Koch[Tetrapanax papyriferus],12g		
	Huangqi,30g	Astragalus membranaceus (Fisch.) Bunge. [Astragalus membranaceus],10g		
	Xiebai,12g	*Amaryllidaceae* J. St.-Hil.[*Allium macrostemon* Bunge],12g		
	Chuanniuxi ,10g	*Cyathula officinalis* Kuan[Amaranthaceae;Radix Cyathulae],10g		
ZLi 2022[13]	Baishao,10g	Cynanchum otophyllum Schneid.[Contortae.],10g	N	N
	Xixin,10g	Asarum heterotropoides F. Schmidt [Aristolochiaceae Juss.],10g		
	Danggui,12g	Angelicae Sinensis Radix [Umbelliferae](Danggui),10g		
	Gancao,8g	Glycyrrhiza uralensis Fisch.[Fabaceae Lindl.],8g		
	Guizhi,10g	Cinnamomum cassia (L.) D.[DonRamulus Cinnamomi],10g		

	Tongcao,12g	Tetrapanax papyrifer (Hook.) K. Koch[Tetrapanax papyriferus],12g		
	Shengma,10g	Cimicifuga foetida L.[Ranunculaceae;Cimicifugae Rhizoma],10 g		
	Zexie,10g	Alisma orientalis(Sam.)Juzep.[Alismataceae;Alismatis Rhizoma],10 g		
	Shengjiang,10g	Zingiberaceae.[Zingiber officinale Roscoe],10g		
HDu 2022[14]	Danggui,10g	Angelicae Sinensis Radix [Umbelliferae](Danggui),10g	N	N
	Xixin,10g	Asarum heterotropoides F. Schmidt [Aristolochiaceae Juss.],10g		
	Baishao,10g	Cynanchum otophyllum Schneid.[Contortae.],10g		
	Gancao,8g	Glycyrrhiza uralensis Fisch.[Fabaceae Lindl.],8g		
	Guizhi,10g	Cinnamomum cassia (L.) D.[DonRamulus Cinnamomi],10g		

	Tongcao,12g	Tetrapanax papyrifer (Hook.) K. Koch[Tetrapanax papyriferus],12g		
	Huangqi,30g	Astragalus membranaceus (Fisch.) Bunge. [Astragalus membranaceus],10g		
	Xiebai,12g	Amaryllidaceae J. St.-Hil.[Allium macrostemon Bunge],12g		
	Chuanniuxi ,10g	Cyathula officinalis Kuan[Amaranthaceae;Radix Cyathulae],10g		
	Danggui,10g	Angelica sinensis (Oliv.) Diels [Umbelliferae;Angelicae Sinensis Radix],10g		
	Xixin,10g	Asarum heterotropoides F. Schmidt [Aristolochiaceae Juss.],10g		
JCui 2021[15]	Danggui,10g	Angelicae Sinensis Radix [Umbelliferae](Danggui),10g	N	N
	Xixin,10g	Asarum heterotropoides F. Schmidt [Aristolochiaceae Juss.],10g		
	Baishao,10g	Cynanchum otophyllum Schneid.[Contortae.],10g		
	Gancao,8g	Glycyrrhiza uralensis Fisch.[Fabaceae Lindl.],8g		

	Guizhi,10g	Cinnamomum cassia (L.) D.[DonRamulus Cinnamomi],10g		
	Tongcao,12g	Tetrapanax papyrifer (Hook.) K. Koch[Tetrapanax papyriferus],12g		
	Huangqi,30g	Astragalus membranaceus (Fisch.) Bunge. [Astragalus membranaceus],10g		
	Xiebai,12g	Amaryllidaceae J. St.-Hil.[Allium macrostemon Bunge],12g		
	Chuanniuxi ,10g	Cyathula officinalis Kuan[Amaranthaceae;Radix Cyathulae],10g		
	Renshen,15g	Araliaceae Juss..[Panax ginseng C. A. Mey],15g		
CWang 2021[16]	Danggui,10g	Angelicae Sinensis Radix [Umbelliferae](Danggui),10g	N	N
	Xixin,10g	Asarum heterotropoides F. Schmidt [Aristolochiaceae Juss.],10g		
	Baishao,10g	Cynanchum otophyllum Schneid.[Contortae.],10g		
	Gancao,8g	Glycyrrhiza uralensis Fisch.[Fabaceae Lindl.],8g		

	Guizhi,10g	Cinnamomum cassia (L.) D.[DonRamulus Cinnamomi],10g		
	Tongcao,12g	Tetrapanax papyrifer (Hook.) K. Koch[Tetrapanax papyriferus],12g		
	Huangqi,30g	Astragalus membranaceus (Fisch.) Bunge. [Astragalus membranaceus],10g		
	Xiebai,12g	Amaryllidaceae J. St.-Hil.[Allium macrostemon Bunge],12g		
	Chuanniuxi ,10g	Cyathula officinalis Kuan[Amaranthaceae;Radix Cyathulae],10g		
	Renshen,15g	Araliaceae Juss..[Panax ginseng C. A. Mey],15g		
	Chaihu,6g	Apiaceae Lindl..[Bupleurum chinense DC.],6g		
	Yujin,3g	Scutellaria baicalensis Georgi.[Curcuma aromatica Salisb],3g		
	Hehuanpi,6g	Radix Puerariae Lobatae.[Legumin0sae],6g		
DWang	Danggui,10g	Angelicae Sinensis Radix [Umbelliferae](Danggui),10g	N	N

2021[17]	Xixin,10g	Asarum heterotropoides F. Schmidt [Aristolochiaceae Juss.],10g		
	Baishao,10g	Cynanchum otophyllum Schneid.[Contortae.],10g		
	Gancao,8g	Glycyrrhiza uralensis Fisch.[Fabaceae Lindl.],8g		
	Guizhi,10g	Cinnamomum cassia (L.) D.[DonRamulus Cinnamomi],10g		
	Tongcao,12g	Tetrapanax papyrifer (Hook.) K. Koch[Tetrapanax papyriferus],12g		
	Huangqi,30g	Astragalus membranaceus (Fisch.) Bunge. [Astragalus membranaceus],10g		
	Xiebai,12g	Amaryllidaceae J. St.-Hil.[Allium macrostemon Bunge],12g		
	Chuanniuxi ,10g	Cyathula officinalis Kuan[Amaranthaceae;Radix Cyathulae],10g		
	Renshen,15g	Apiales， Umbellales..[Panax ginseng C. A. Mey],15g		
XJia 2021[18]	Danggui,10g	Angelicae Sinensis Radix [Umbelliferae](Danggui),10g	N	N

	Xixin,10g	Asarum heterotropoides F. Schmidt [Aristolochiaceae Juss.],10g		
	Baishao,10g	Cynanchum otophyllum Schneid.[Contortae.],10g		
	Gancao,8g	Glycyrrhiza uralensis Fisch.[Fabaceae Lindl.],8g		
	Guizhi,10g	Cinnamomum cassia (L.) D.[DonRamulus Cinnamomi],10g		
	Tongcao,12g	Tetrapanax papyrifer (Hook.) K. Koch[Tetrapanax papyriferus],12g		
	Huangqi,30g	Astragalus membranaceus (Fisch.) Bunge. [Astragalus membranaceus],10g		
	Xiebai,12g	Amaryllidaceae J. St.-Hil.[Allium macrostemon Bunge],12g		
	Chuanniuxi ,10g	Cyathula officinalis Kuan[Amaranthaceae;Radix Cyathulae],10g		
	Chaihu,6g	Apiaceae Lindl..[Bupleurum chinense DC.],6g		
QYuan	Danggui,10g	Angelicae Sinensis Radix [Umbelliferae](Danggui),10g	N	N

2020[19]	Xixin,10g	Asarum heterotropoides F. Schmidt [Aristolochiaceae Juss.],10g		
	Baishao,10g	Cynanchum otophyllum Schneid.[Contortae.],10g		
	Gancao,8g	Glycyrrhiza uralensis Fisch.[Fabaceae Lindl.],8g		
	Guizhi,10g	Cinnamomum cassia (L.) D.[DonRamulus Cinnamomi],10g		
	Tongcao,12g	Tetrapanax papyrifer (Hook.) K. Koch[Tetrapanax papyriferus],12g		
	Huangqi,30g	Astragalus membranaceus (Fisch.) Bunge. [Astragalus membranaceus],10g		
	Xiebai,12g	Amaryllidaceae J. St.-Hil.[Allium macrostemon Bunge],12g		
	Chuanniuxi ,10g	Cyathula officinalis Kuan[Amaranthaceae;Radix Cyathulae],10g		
	Renshen,15g	Araliaceae Juss..[Panax ginseng C. A. Mey],15g		
EChen	Danggui,10g	Angelicae Sinensis Radix [Umbelliferae](Danggui),10g	N	N

2020[20]	Xixin,10g	Asarum heterotropoides F. Schmidt [Aristolochiaceae Juss.],10g		
	Baishao,10g	Cynanchum otophyllum Schneid.[Contortae.],10g		
	Gancao,8g	Glycyrrhiza uralensis Fisch.[Fabaceae Lindl.],8g		
	Guizhi,10g	Cinnamomum cassia (L.) D.[DonRamulus Cinnamomi],10g		
	Tongcao,12g	Tetrapanax papyrifer (Hook.) K. Koch[Tetrapanax papyriferus],12g		
	Huangqi,30g	Astragalus membranaceus (Fisch.) Bunge. [Astragalus membranaceus],10g		
	Xiebai,15g	Amaryllidaceae J. St.-Hil.[Allium macrostemon Bunge],15g		
	Chuanniuxi ,8g	Cyathula officinalis Kuan[Amaranthaceae;Radix Cyathulae],8g		
XChen 2019[21]	Danggui,10g	Angelicae Sinensis Radix [Umbelliferae](Danggui),10g	N	N
	Xixin,10g	Asarum heterotropoides F. Schmidt [Aristolochiaceae Juss.],10g		

	Baishao,10g	Cynanchum otophyllum Schneid.[Contortae.],10g		
	Gancao,8g	Glycyrrhiza uralensis Fisch.[Fabaceae Lindl.],8g		
	Guizhi,10g	Cinnamomum cassia (L.) D.[DonRamulus Cinnamomi],10g		
	Tongcao,12g	Tetrapanax papyrifer (Hook.) K. Koch[Tetrapanax papyriferus],12g		
	Huangqi,30g	Astragalus membranaceus (Fisch.) Bunge. [Astragalus membranaceus],10g		
	Xiebai,12g	Amaryllidaceae J. St.-Hil.[Allium macrostemon Bunge],12g		
	Chuanniuxi ,10g	Cyathula officinalis Kuan[Amaranthaceae;Radix Cyathulae],10g		
	Renshen,15g	Araliaceae Juss..[Panax ginseng C. A. Mey],15g		
	Chaihu,6g	Apiaceae Lindl..[Bupleurum chinense DC.],6g		

	Yujin,3g	Scutellaria baicalensis Georgi.[Curcuma aromatica Salisb],3g		
	Hehuanpi,6g	Radix Puerariae Lobatae.[Legumin0sae],6g		
ZXu 2019[22]	Danggui,10g	Angelicae Sinensis Radix [Umbelliferae](Danggui),10g	N	N
	Xixin,10g	Asarum heterotropoides F. Schmidt [Aristolochiaceae Juss.],10g		
	Baishao,10g	Cynanchum otophyllum Schneid.[Contortae.],10g		
	Gancao,8g	Glycyrrhiza uralensis Fisch.[Fabaceae Lindl.],8g		
	Guizhi,10g	Cinnamomum cassia (L.) D.[DonRamulus Cinnamomi],10g		
	Tongcao,12g	Tetrapanax papyrifer (Hook.) K. Koch[Tetrapanax papyriferus],12g		
	Huangqi,30g	Astragalus membranaceus (Fisch.) Bunge. [Astragalus membranaceus],10g		
	Xiebai,12g	Amaryllidaceae J. St.-Hil.[Allium macrostemon Bunge],12g		

	Chuanniuxi ,10g	Cyathula officinalis Kuan[Amaranthaceae;Radix Cyathulae],10g		
	Renshen,15g	Araliaceae Juss..[Panax ginseng C. A. Mey],15g		
	Chaihu,6g	Apiaceae Lindl..[Bupleurum chinense DC.],6g		
	Shengma,5g	*Cimicifuga foetida* L.[Ranunculaceae;Cimicifugae Rhizoma],5g		
LLu 2019[23]	Danggui,10g	Angelicae Sinensis Radix [Umbelliferae](Danggui),10g	N	N
	Xixin,10g	Asarum heterotropoides F. Schmidt [Aristolochiaceae Juss.],10g		
	Baishao,10g	Cynanchum otophyllum Schneid.[Contortae.],10g		
	Gancao,8g	Glycyrrhiza uralensis Fisch.[Fabaceae Lindl.],8g		
	Guizhi,10g	Cinnamomum cassia (L.) D.[DonRamulus Cinnamomi],10g		
	Tongcao,12g	Tetrapanax papyrifer (Hook.) K. Koch[Tetrapanax papyriferus],12g		

	Huangqi,30g	Astragalus membranaceus (Fisch.) Bunge. [Astragalus membranaceus],10g		
	Xiebai,12g	Amaryllidaceae J. St.-Hil.[Allium macrostemon Bunge],12g		
	Chuanniuxi ,10g	Cyathula officinalis Kuan[Amaranthaceae;Radix Cyathulae],10g		
	Renshen,15g	Araliaceae Juss..[Panax ginseng C. A. Mey],15g		
	Chaihu,6g	Apiaceae Lindl..[Bupleurum chinense DC.],6g		
	Yujin,3g	Scutellaria baicalensis Georgi.[Curcuma aromatica Salisb],3g		
	Hehuanpi,6g	Radix Puerariae Lobatae.[Legumin0sae],6g		
HZhang 2017[24]	Baishao,10g	Cynanchum otophyllum Schneid.[Contortae.],10g	N	N
	Xixin,10g	Asarum heterotropoides F. Schmidt [Aristolochiaceae Juss.],10g		
	Danggui,12g	Angelicae Sinensis Radix [Umbelliferae](Danggui),10g		

	Gancao,8g	Fabaceae Lindl..[Glycyrrhiza uralensis Fisch.],8g		
	Guizhi,10g	Cinnamomum cassia (L.) D.[DonRamulus Cinnamomi],10g		
	Tongcao,12g	Tetrapanax papyrifer (Hook.) K. Koch[Tetrapanax papyriferus],12g		
	Shengma,10g	Cimicifuga foetida L.[Ranunculaceae;Cimicifugae Rhizoma],10 g		
	Zexie,10g	Alisma orientalis(Sam.)Juzep.[Alismataceae;Alismatis Rhizoma],10 g		
	Chaihu,6g	Apiaceae Lindl..[Bupleurum chinense DC.],6g		
	Renshen,15g	Araliaceae Juss..[Panax ginseng C. A. Mey],15g		
	Chaihu,6g	Apiaceae Lindl..[Bupleurum chinense DC.],6g		
	Yujin,3g	Scutellaria baicalensis Georgi.[Curcuma aromatica Salisb],3g		
	dān shēn,10g	*Salvia miltiorrhiza* Bunge [Lamiaceae; Salviae miltiorrhizae radix et rhizoma],10g		

Supplementary File S1 The PRISMA checklist of this meta-analysis

Section and Topic	Item #	Checklist item	Location where item is reported
**TITLE**			
Title	1	Identify the report as a systematic review.	
**ABSTRACT**			
Abstract	2	See the PRISMA 2020 for Abstracts checklist.	
**INTRODUCTION**			
Rationale	3	Describe the rationale for the review in the context of existing knowledge.	
Objectives	4	Provide an explicit statement of the objective(s) or question(s) the review addresses.	
**METHODS**			
Eligibility criteria	5	Specify the inclusion and exclusion criteria for the review and how studies were grouped for the syntheses.	
Information sources	6	Specify all databases, registers, websites, organisations, reference lists and other sources searched or consulted to identify studies. Specify the date when each source was last searched or consulted.	
Search strategy	7	Present the full search strategies for all databases, registers and websites, including any filters and limits used.	
Selection process	8	Specify the methods used to decide whether a study met the inclusion criteria of the review, including how many reviewers screened each record and each report retrieved, whether they worked independently, and if applicable, details of automation tools used in the process.	
Data collection process	9	Specify the methods used to collect data from reports, including how many reviewers collected data from each report, whether they worked independently, any processes for obtaining or confirming data from study investigators, and if applicable, details of automation tools used in the process.	

Section and Topic	Item #	Checklist item	Location where item is reported
Data items	10a	List and define all outcomes for which data were sought. Specify whether all results that were compatible with each outcome domain in each study were sought (e.g. for all measures, time points, analyses), and if not, the methods used to decide which results to collect.	
	10b	List and define all other variables for which data were sought (e.g. participant and intervention characteristics, funding sources). Describe any assumptions made about any missing or unclear information.	
Study risk of bias assessment	11	Specify the methods used to assess risk of bias in the included studies, including details of the tool(s) used, how many reviewers assessed each study and whether they worked independently, and if applicable, details of automation tools used in the process.	
Effect measures	12	Specify for each outcome the effect measure(s) (e.g. risk ratio, mean difference) used in the synthesis or presentation of results.	
Synthesis methods	13a	Describe the processes used to decide which studies were eligible for each synthesis (e.g. tabulating the study intervention characteristics and comparing against the planned groups for each synthesis (item #5)).	
	13b	Describe any methods required to prepare the data for presentation or synthesis, such as handling of missing summary statistics, or data conversions.	
	13c	Describe any methods used to tabulate or visually display results of individual studies and syntheses.	
	13d	Describe any methods used to synthesize results and provide a rationale for the choice(s). If meta-analysis was performed, describe the model(s), method(s) to identify the presence and extent of statistical heterogeneity, and software package(s) used.	
	13e	Describe any methods used to explore possible causes of heterogeneity among study results (e.g. subgroup analysis, meta-regression).	
	13f	Describe any sensitivity analyses conducted to assess robustness of the synthesized results.	
Reporting bias assessment	14	Describe any methods used to assess risk of bias due to missing results in a synthesis (arising from reporting biases).	

Section and Topic	Item #	Checklist item	Location where item is reported
Certainty assessment	15	Describe any methods used to assess certainty (or confidence) in the body of evidence for an outcome.	
**RESULTS**			
Study selection	16a	Describe the results of the search and selection process, from the number of records identified in the search to the number of studies included in the review, ideally using a flow diagram.	
	16b	Cite studies that might appear to meet the inclusion criteria, but which were excluded, and explain why they were excluded.	
Study characteristics	17	Cite each included study and present its characteristics.	
Risk of bias in studies	18	Present assessments of risk of bias for each included study.	
Results of individual studies	19	For all outcomes, present, for each study: (a) summary statistics for each group (where appropriate) and (b) an effect estimate and its precision (e.g. confidence/credible interval), ideally using structured tables or plots.	
Results of syntheses	20a	For each synthesis, briefly summarise the characteristics and risk of bias among contributing studies.	
	20b	Present results of all statistical syntheses conducted. If meta-analysis was done, present for each the summary estimate and its precision (e.g. confidence/credible interval) and measures of statistical heterogeneity. If comparing groups, describe the direction of the effect.	
	20c	Present results of all investigations of possible causes of heterogeneity among study results.	
	20d	Present results of all sensitivity analyses conducted to assess the robustness of the synthesized results.	
Reporting biases	21	Present assessments of risk of bias due to missing results (arising from reporting biases) for each synthesis assessed.	

Section and Topic	Item #	Checklist item	Location where item is reported
Certainty of evidence	22	Present assessments of certainty (or confidence) in the body of evidence for each outcome assessed.	
**DISCUSSION**			
Discussion	23a	Provide a general interpretation of the results in the context of other evidence.	
	23b	Discuss any limitations of the evidence included in the review.	
	23c	Discuss any limitations of the review processes used.	
	23d	Discuss implications of the results for practice, policy, and future research.	
**OTHER INFORMATION**			
Registration and protocol	24a	Provide registration information for the review, including register name and registration number, or state that the review was not registered.	
	24b	Indicate where the review protocol can be accessed, or state that a protocol was not prepared.	
	24c	Describe and explain any amendments to information provided at registration or in the protocol.	
Support	25	Describe sources of financial or non-financial support for the review, and the role of the funders or sponsors in the review.	
Competing interests	26	Declare any competing interests of review authors.	
Availability of data, code and other materials	27	Report which of the following are publicly available and where they can be found: template data collection forms; data extracted from included studies; data used for all analyses; analytic code; any other materials used in the review.	

From: Page MJ, McKenzie JE, Bossuyt PM, Boutron I, Hoffmann TC, Mulrow CD, et al. The PRISMA 2020 statement: an updated guideline for reporting systematic reviews. BMJ 2021;372:n71. doi: 10.1136/bmj.n71

For more information, visit: http://www.prisma-statement.org/

Supplementary File S2 GRADE quality of evidence

Outcomes	Number of RCTs	Evidence quality evaluation					Sample size		Effect	Level of Evidence
		Boundedness	Inconsistency	Indirection	Inaccuracy	Publication bias	Experimental group	control group		
Efficacy in angina pectoris	8	Serious (1)	Not serious	Not serious	Not serious	Not found	285/318（90.4%）	232/318（72.9%）	RR=1.23, 95% CI [1.14, 1.33]	B
Effective rate of TCM symptoms	7	Serious (1)	Not serious	Not serious	Not serious	Not found	331/364（90.1%）	277/364（70.1%）	RR=1.19, 95% CI [1.12, 1.28]	B
Nitroglycerin usage reduction	4	Serious (1)	Not serious	Not serious	Serious (3)	Not found	114/135（84.4%）	89/135（70.2%）	RR=1.28, 95% CI [1.11, 1.47]	C
ECG effectiveness rate	4	Serious (1)	Not serious	Not serious	Serious (3)	Not found	100/135（74.1%）	79/135（58.1%）	RR=1.27, 95% CI [1.06, 1.51]	C
Number of angina attacks	10	Serious (1)	Serious (2)	Not serious	Not serious	Not found	508	508	MD=-1.87, 95% CI [-2.63, -1.10]	C
Duration of angina	10	Serious (1)	Serious (2)	Not serious	Not serious	Not found	508	508	MD=-1.78, 95% CI	C

pectoris									[-2.16, -1.39]	
Indicators of cardiac function	5	Serious (1)	Serious (2)	Not serious	Not serious	Not found	827	827	MD=0.31, 95% CI [-0.47, 1.10]	C
TCM symptom score	5	Serious (1)	Not serious	Not serious	Not serious	Not found	196	196	MD=-2.90, 95% CI [-3.47, -2.34]	B
Seattle Angina Questionnaire	3	Serious (1)	Serious (2)	Not serious	Serious (3)	Not found	136	136	MD=8.31, 95% CI [7.35, 9.27]	D
NT-ProBNP	2	Serious (1)	Not serious	Not serious	Serious (3)	Not found	78	78	MD=-333.63, 95% CI [-362.00, -305.25]	C
Inflammatory factors	2	Serious (1)	Not serious	Not serious	Serious (3)	Not found	78	78	MD=-5.45, 95% CI[-5.91, - 4.98]	C
Adverse events rates	2	Serious (1)	Not serious	Not serious	Serious (3)	Not found	180	180	OR=0.23, 95% CI [0.06, 0.92]	C

Note: (1) The risk of bias is reduced by one level: Some studies have unclear reports on randomized methods, allocation concealment, and blind methods; (2) Reduce the risk of inconsistency by one level: there is inconsistency in the results without a reasonable explanation; (3) The risk of imprecision is reduced by one level: the relatively small sample size leads to a wider confidence interval, which affects accuracy;
